# Trend and projection of larynx cancer incidence and mortality in China from 1990 to 2044: A Bayesian age–period–cohort modeling study

**DOI:** 10.1002/cam4.6239

**Published:** 2023-06-12

**Authors:** Enlin Ye, Jiasheng Huang, Jia Wang, Yumei Zhao, Dongdong Niu, Jie Liu, Xueying Huang, Suru Yue, Xuefei Hou, Jiayuan Wu

**Affiliations:** ^1^ Clinical Research Service Center Affiliated Hospital of Guangdong Medical University Zhanjiang China; ^2^ Guangdong Engineering Research Center of Collaborative Innovation Technology of Clinical Medical Big Data Cloud Service in Medical Consortium of West Guangdong Province Affiliated Hospital of Guangdong Medical University Zhanjiang China

**Keywords:** age–period–cohort model, deaths, incidence, joinpoint regression analysis, larynx cancer

## Abstract

**Background:**

Larynx cancer is one of the most common cancers in head and neck, and imposes heavy burden on individual and societies. A comprehensive understanding of the burden of larynx cancer is necessary to improve prevention and control strategies. However, the secular trend of larynx cancer incidence and mortality in China remains unclear.

**Methods:**

The incidence and deaths rates of larynx cancer from 1990 to 2019 were collected from the Global Burden of Disease Study 2019 database. The temporal trend of larynx cancer was analyzed using a joinpoint regression model. The age–period–cohort model was used to explore the age, period, and cohort effects on larynx cancer and predict future trends up to 2044.

**Results:**

From 1990 to 2019, the age‐standardized incidence rate of larynx cancer in China increased by 1.3% (95% CI 1.1 to 1.5) in males, but decreased by 0.5% (95% CI −0.1 to 0) in females. The age‐standardized mortality rate of larynx cancer in China decreased by 0.9% (95% CI −1.1 to −0.6) and 2.2% (95% CI −2.8 to −1.7) in males and females, respectively. Among the four risk factors, smoking and alcohol use contributed to a heavier burden compared to occupational exposure to asbestos and sulfuric acid with respect to mortality. Age effects showed that the incidence and deaths of larynx cancer were concentrated in people older than 50 years old. Period effects exerted the most significant effect on larynx cancer incidence for males. In terms of cohort effects, people born in the earlier cohorts presented a higher risk of larynx cancer compared with the later cohorts. From 2020 to 2044, the age‐standardized incidence rates of larynx cancer continued to increase in males, whereas the age‐standardized mortality rates continued to decrease in both males and females.

**Conclusion:**

The burden of larynx cancer in China has a significant gender difference. The age‐standardized incidence rates will continue to increase in males up to 2044. The disease pattern and risk factors of larynx cancer should be comprehensively studied to promote the development of timely intervention measures and relieve the burden effectively.

## 1 INTRODUCTION

Larynx cancer is a common malignant tumor in the head and neck, and its main histopathological form is laryngeal squamous cell carcinoma which is insensitive to conventional chemotherapy.^1^ Therefore, surgery combined with radiotherapy or chemotherapy has been the most common treatment method for larynx cancer.^2^ The prognosis of larynx carcinoma is closely related to the primary location, invasion range, pathological classification, differentiation degree, lymph node metastasis, and clinical therapy. The survival rate and quality of life of patients with early larynx cancer can be significantly improved after surgery and radiotherapy, but the 5‐year survival rate of advanced larynx cancer remains low.^1^

Larynx cancer accounts for 7.9%–35% of malignant tumors in otolaryngology, ranking third among malignant tumors in the neck; it causes great harm to the life and health of residents, notably males, in the worldwide.^3^ According to the Global Burden of Disease study (GBD) 2017, the incidence and mortality of larynx cancer decreased significantly in the world and most countries from 1990 to 2017.^4^ Over the past 30 years, only 3 out of 21 GBD regions have seen a significant increase in age‐standardized incidence rate, namely East Asia, the Caribbean, and Oceania.^3^ However, the incidence and mortality rates of larynx cancer in China are lower than the world average, and China accounted for 0.67% and 0.57% of all new cases and deaths from larynx cancer globally in 2019, respectively.^5^

The risk of larynx cancer is significantly related to smoking, alcohol use, sex hormones, human papillomavirus (HPV) infection, air pollution, and occupational exposure factors (including asbestos, sulfuric acid, and mustard gas), which may vary with chronological age, time period, and birth cohort.^6^, ^7^, ^8^, ^9^, ^10^ However, few studies have explored the age, period, and cohort effects on the disease burden of larynx cancer. Traditional time‐series analysis mainly focuses on some single variables, such as age‐specific incidence rate, which only considers the impact of age on the incidence of chronic disease, and ignores the influence of the interrelationship between different periods, birth cohorts, and age on the burden of chronic disease. At the same time, in many time‐series studies, scholars only pay attention to the diachronic changes (the period effect) and ignore the complexity of social changes in the time dimension. In terms of time dimension, social change includes three mutual “confounding” effects of age, period, and birth cohort, and these three factors can produce mutual restricting effects. Therefore, it is necessary to investigate these three effects simultaneously in an analytical framework to more accurately understand the changes of a specific time dimension and their respective interpretation mechanisms. Age–period–cohort analysis has been a common method used to assess the contributions of age, period, and cohort effects on the outcome. Age effect reveals the differences in biological or social processes related to age. Period effect reflect the effects of the complex combination of historical events and environmental factors that affect all age groups. Cohort effect emphasizes the historical differences between groups born in different times.^11^ Compared with traditional analysis methods, the age–period–cohort models can be carried out under the condition of simultaneously adjusting and controlling the three factors of age, period, and cohort. The present work aimed to analyze the temporal trends of larynx cancer incidence and deaths in China based on the data from the GBD 2019 study and to explore the age, period, and cohort effects under the age–period–cohort framework. Moreover, we used the Bayesian age–period–cohort (BAPC) model to predict the incidence and mortality of larynx cancer in China from 2020 to 2044. The results of this study may provide important clues or hypotheses for studying disease burden and its trend changes and exploring the etiology of the larynx cancer, which not only contributes to improve public health at the national level, allocate medical resources reasonably, and provide a basis for formulating health strategies, but also helps to measure the decision of specific diagnosis and treatment.

## 2 MATERIALS AND METHODS

### 2.1 Data sources

Data on incidence and deaths rates of larynx cancer in China were obtained from the official website of GBD 2019 (http://ghdx.healthdata.org/gbdresults‐tool).^12^ The GBD 2019 quantifies the incidence, prevalence, deaths, and disability‐adjusted life‐years of 369 diseases and injuries for 204 countries and territories from 1990 to 2019.^13^ Details of the GBD 2019 case definition, input data, and modeling strategy were reported in previous studies.^14^ The data used in the GBD 2019 were obtained from literature reports, epidemiological surveillance, hospital discharge, outpatient visits, and medical insurance claim records.^15^ The ICD‐10 codes (C32–C32.9, D02.0, D14.1, and D38.0) and ICD‐9 codes (161–161.9, 212.1, 231.0, and 235.6) were used to identify larynx cancer. We extracted the age‐standardized rates (ASRs) of incidence and mortality by sex and year, as well as age‐standardized mortality rate and percent of larynx cancer attributable to four risk factors (smoking, alcohol use, occupational exposure to asbestos and sulfuric acid) to reflect the secular trend of larynx cancer burden in China. The rough sum of the percentage of deaths caused by risk factors may exceed 100%, as many risk factors are partially or entirely mediated by another or more risk factors.^16^ In GBD, the degree of uncertainty about the estimates was expressed by uncertainty interval (UI). ASRs and 95% UI were calculated based on the GBD 2019 global age‐standard population. Moreover, sex‐specific crude rates and 95% UIs of different age groups were obtained.

### 2.2 Joinpoint regression analysis

Joinpoint Regression Program (4.7.0.0.) software developed by the United States National Cancer Institute is used for trend analysis of the incidence and deaths rates over time by using connected linear segments on a logarithmic scale.^17^ The best fitting point is called the joinpoint, and the location of statistically significant change is determined by joinpoint analysis.^18^ We used the calendar year as a regression variable to fit the regression line to the natural logarithm of the observed rates.

The expression of joinpoint regression model is generally written as:

$$E\left[y/x\right]=\beta_{0}+\beta_{1}x+\delta_{1}{\left(x-\tau_{1}\right)}^{+}+\cdots +\delta_{k}{\left(x-\tau_{k}\right)}^{+}$$

From the expression, *y* is the dependent variable in the model, that is, morbidity or mortality; *x* is the independent variable in the model, that is, the calendar year; *β* represents constant terms in the model; *δ*_*i*_ (*i* = 1,2,…, *k*) represents the regression coefficient for each segmentation function; *τ*_*i*_ (*i* = 1, 2,…, *k*) represents turning points; and *k* represents the number of turning points.

The regression model was established to calculate annual percent change (APC), average annual percent change (AAPC), and the corresponding 95% confidence interval (CI), which can reflect the trend of each segment in the observation period. If AAPC or APC > 0 with its 95% CI not exceeding 0, the ASRs of the corresponding period increases year by year; by contrast, if AAPC or APC < 0 with its 95% CI does not exceed 0, it denotes decreases year by year. Otherwise, the ASRs were regarded as stable over time.

### 2.3 Age–period–cohort analysis

The age–period–cohort model based on Poisson distribution can simultaneously estimate the net effect of age, period, and cohort on disease burden, which goes beyond the traditional epidemiological analysis.^19^

The expression of age–period–cohort model is generally written as:

$$Y=\log\left(m\right)=\mu +\alpha \times \mathrm{age}1+\beta \times \text{period}1+\gamma \times \text{cohort}1+\varepsilon$$

From the expression, *M* stand for the rates of the corresponding age group, *μ* stands for the intercept item, *α*, *β*, and *γ* stand for the age, period and cohort effect, and *ε* is the random error.

The age–period–cohort model requires an equal time interval in age, period, and cohort. In the GBD 2019 database, the incidence and mortality rates of larynx cancer were not recorded in the population aged 0 to 19 years, thus, the age analysis was started at 20 years old. Meanwhile, the age group of 95 years plus was excluded from the analysis because its age grouping did not satisfy the data format of the age–period–cohort model. Therefore, the age groups were divided into 15 groups (20 to 24, 25 to 29, …, 90 to 94 years). The classification by age was matched by further organizing data by six period cohorts (1994, 1999, 2004, 2009, 2014, and 2019) and 20 equal birth cohorts starting from 1904–1909 to 1999–2004.

The non‐identifiability problem (cohort = period − age) leads to difficulty in evaluating of the independent effects of age, period, and cohort.^20^ To solve this problem, the age–period–cohort framework with the intrinsic estimator (IE) method was performed to estimate the coefficients for age, period, and cohort effects.^11^ These coefficients can be converted into exponential values [exp(coef.) = e^coef^] to represent the average level of relative risk (RR) of specific age, period, or birth cohort relative to all ages, periods, or birth cohorts.^21^ For example, the RR of larynx cancer incidence in old people aged 85 to 89 years was 3.26, indicating that the risk in this age group was 3.26 times higher than that in all age groups. The age–period–cohort analysis was performed using STATA 15.0 software.

### 2.4 Projection of incidence and death rates

Based on the trends of larynx cancer burden from 1990 to 2019, our study predicted the ASRs of incidence and death rates from 2020 to 2044 by using the BAPC model. The BAPC model was operated by the INLA package in R (version 4.1.2), using a sex‐specific APC model. A Poisson process was performed to build a model based on the number of cases (*y*_*ijs*_) for each age group (*i*), period (*j*), and sex (*s*); the mean of which is the product of the population at risk (*N*_*ijs*_) and the estimated rate. The log of the rate (*η*_*ijs*_) was estimated as a linear combination of a sex‐specific intercept (*μ*_*s*_), an overdispersion effect (*z*_*ijs*_), the age effects (*α*_*is*_), the period effects (*β*_*js*_), and the cohort effects (*γ*_*ks*_). The birth cohort was evaluated through *k = M(I‐i) + j*, of which *M* denotes the number of periods per age group and *I* denotes the number of age groups.

$$y_{\mathrm{ijs}}∼\text{poisson}\left(N_{\mathrm{ijs}}\exp\left(\eta_{\mathrm{ijs}}\right)\right)$$

$$\eta_{\mathrm{ijs}}=\mu_{s}+\alpha_{\text{is}}+\beta_{\mathrm{js}}+\gamma_{\mathrm{ks}}+Z_{\mathrm{ijs}}$$

Second‐order random walk priors are applicable to the age, period, and cohort effects, and the sum is zero.^22^ Log‐gamma priors are applied to the precision parameters. For the age, period, and cohort effects, the scale and shape parameters are 1 and 0.00005, respectively, while for the overdispersion effect, the scale and shape parameters are 1 and 0.005. Overdispersion parameters are given Gaussian priors with a mean value of zero. The posterior distributions of the age‐standardized incidence and deaths rates were calculated using the 2019 China standard population, and projected rates were calculated using the projected China population.

## 3 RESULTS

### 3.1 Descriptive analysis

**FIGURE 1:**
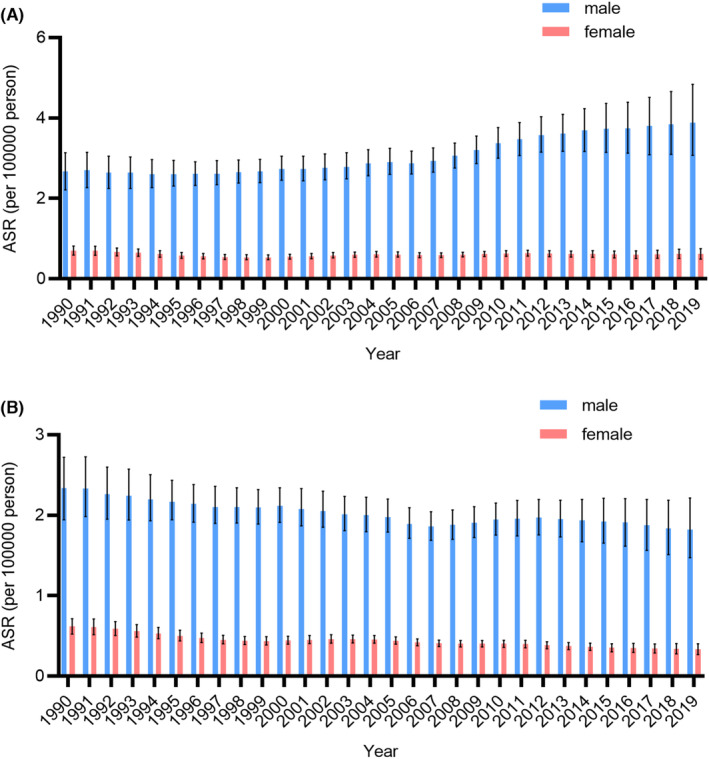
The trends of larynx cancer burden by sex in China from 1990 to 2019. (A) age‐standardized incidence rates; (B) age‐standardized mortality rates. ASR, age‐standardized rates.

Figure 1 shows the sex‐specific ASRs of the incidence and mortality rates of larynx cancer in China from 1990 to 2019. In general, the sex‐specific age‐standardized mortality rates of larynx cancer in males and females showed a downward trend from 1990 to 2019. However, age‐standardized incidence rates of larynx cancer in males showed an upward trend from 1990 to 2019, but those in females remained stable. During the observation period, the age‐standardized incidence rate of larynx cancer in males was significantly higher than that in females.

### 3.2 Joinpoint regression analysis

**FIGURE 2:**
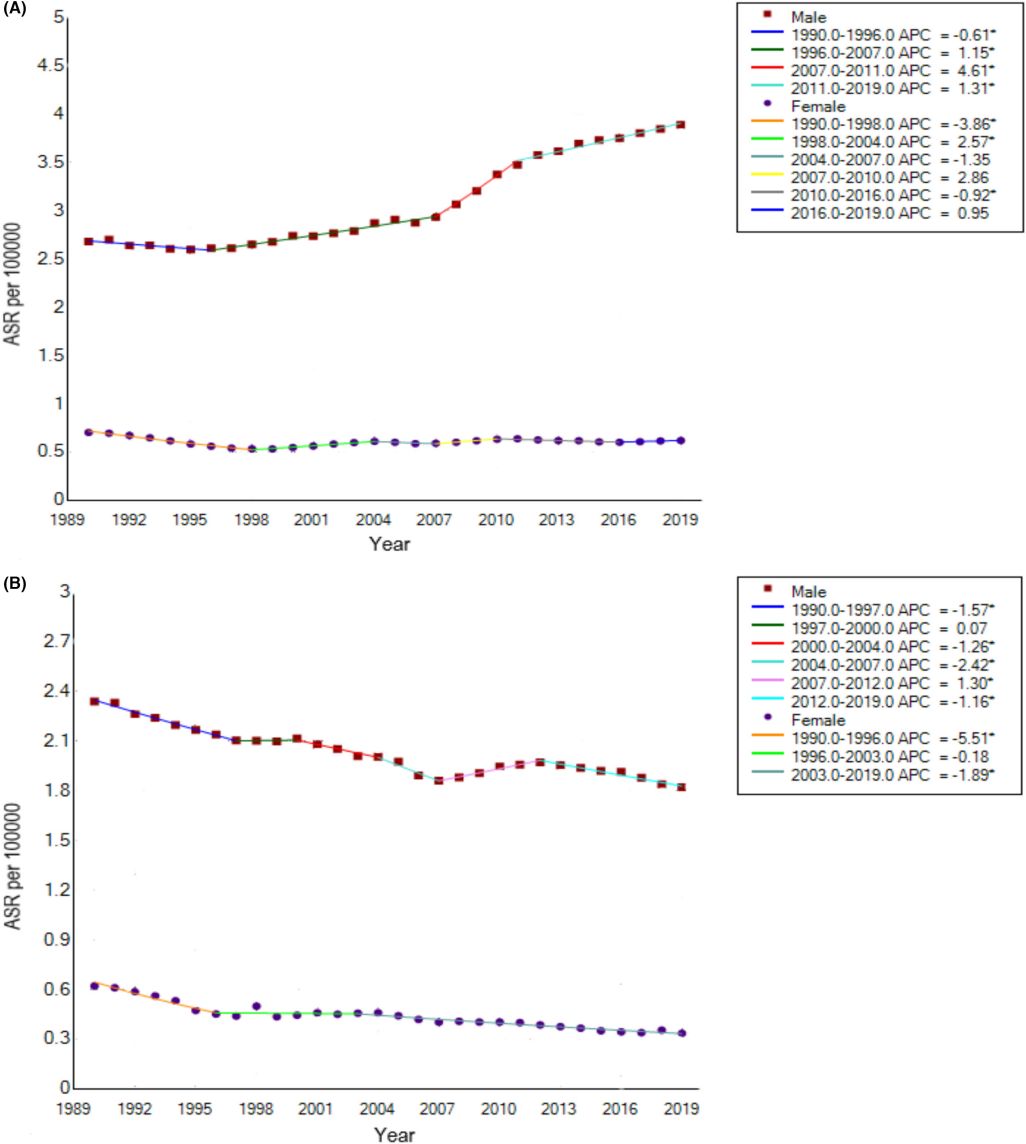
Joinpoint regression analysis in sex‐specific age‐standardized incidence rate (A) and age‐standardized mortality rate (B) of larynx cancer in China from 1990 to 2019. ASR, age‐standardized rates.

The Joinpoint regression analysis results of age–sex‐specific rates of larynx cancer are shown in Figure 2. From 1990 to 2019, the age‐standardized incidence rates of larynx cancer in China increased by 1.3% (95% CI: 1.1 to 1.5) in males, whereas decreased by 0.5% (95% CI: −0.1 to 0) in females. The age‐standardized mortality rates of larynx cancer in China decreased by 0.9% (95% CI: −1.1 to −0.6) in males and 2.2% (95% CI: −2.8 to −1.7) in females. Compared with the males, the females showed substantial decreases in larynx cancer mortality during the past two decades.

### 3.3 Burden attributable to difference risk factors

**FIGURE 3:**
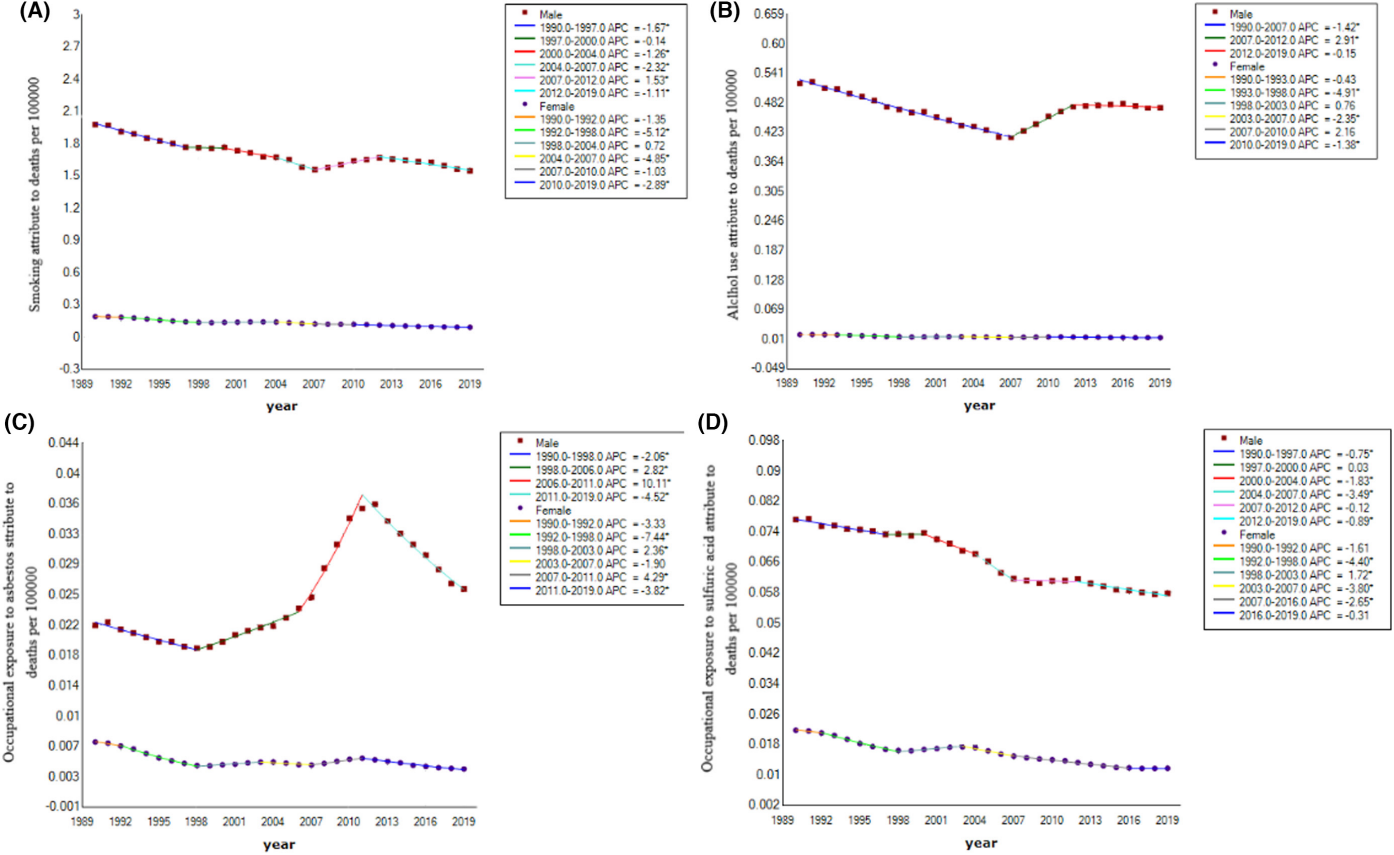
Joinpoint regression analysis in sex‐specific age‐standardized mortality rate of risk factors of larynx cancer in China from 1990 to 2019.(A) mortality rate attributable to smoking (B) mortality rate attributable to alcohol use (C) mortality rate attributable to occupational exposure to asbestos (D) mortality rate attributable to occupational exposure to sulfuric acid.

The Joinpoint regression analysis results of age–sex‐specific mortality rate of four risk factors (smoking, alcohol use, occupational exposure to asbestos and sulfuric acid) of larynx cancer are shown in Figure 3. From 1990 to 2019, the AAPCs of age‐standardized mortality rate attributable to four risk factors (smoking, alcohol use, occupational exposure to asbestos and sulfuric acid) of larynx cancer in China were − 0.8% (95% CI: −1.1 to 0.6), −0.4% (95% CI: −0.5 to −0.2), −1.0% (95% CI: −1.3 to −0.8), and 0.6% (95% CI: 0.3 to 0.8) in males, respectively. During the observation period, the burden of larynx cancer attributable to smoking, alcohol use, occupational exposure to asbestos and sulfuric acid in females showed a continuously decreasing trend, with AAPCs being −2.5% (95% CI: −3.1 to −2.0), −1.3% (95% CI: −1.7 to −0.9), −2.1% (95% CI: −2.4 to −1.8) and − 2.2% (95% CI: −2.7 to −1.7) in females, respectively. From 1990 to 2019, smoking and alcohol use contributed to a heavier burden compared to occupational exposure to asbestos and sulfuric acid with respect to the age‐standardized mortality rates.

Supporting Information Figure S1 showed the proportions of mortality attributable to different risk factors by sex in 2019. For males, 85.01% of the mortality burden of larynx cancer can be attributed to smoking, 25.94% to alcohol use, 1.43% to asbestos, and 3.16% to sulfuric acid. For females, 27.92% of the larynx cancer deaths can be attributed to smoking, 4.01% to alcohol use, 1.18% to asbestos, and 3.51% to sulfuric acid. The proportion of mortality attributable to smoking and alcohol use was much higher in males than that in occupational exposure to asbestos and sulfuric acid.

### 3.4 Age–period–cohort analysis

The RRs of larynx cancer incidence and mortality rates due to age, period, and cohort effects are shown in Tables 1 and 2, respectively. When the period and cohort effects were controlled, the RRs of age effects on larynx cancer incidence rate increased from 0.06 (95% CI: 0.00 to 0.11) in the group aged 20–24 years to 3.61 (95% CI 3.41 to 3.81) in the group aged 85–89 years for males (Supporting Information Figure S2A). For females, the age RRs on larynx cancer incidence increased from 0.16 (95% CI: 0.01 to 0.32) in the group aged 20–24 years to 3.26 (95% CI: 3.07 to 3.45) in the group aged 85–89 years (Supporting Information Figure S2A). The age RRs on larynx cancer mortality rate increased from 0.05 (95% CI: 0.01 to 0.08) in the group aged 20–24 years to 6.07 (95% CI: 5.57 to 6.57) in the group aged 85–89 years for males (Supporting Information Figure S2B). For females, the age RRs on larynx cancer mortality increased with increasing age, from 0.13 (95% CI: 0.02 to 0.25) in the group aged 20–24 years to 5.49 (95% CI: 4.86 to 6.12) in the group aged 90–94 years (Supporting Information Figure S2B).

**TABLE 1:** Sex—specific relative risks of larynx cancer incidence in China due to age, period, and cohort effects.

Factors	Male		Female	
	RR (95% CI)	*p*‐Value	RR (95% CI)	*p*‐Value
Age effects				
20–24	0.06 (0.01–0.11)	<0.001	0.16 (0.01–0.32)	<0.001
25–29	0.07 (0.02–0.13)	<0.001	0.19 (0.01–0.37)	<0.001
30–34	0.11 (0.03–0.20)	<0.001	0.19 (0.01–0.38)	<0.001
35–39	0.25 (0.06–0.45)	<0.001	0.27 (0.07–0.47)	<0.001
40–44	0.54 (0.24–0.84)	0.013	0.37 (0.18–0.56)	<0.001
45–49	1.03 (0.82–1.24)	0.439	0.65 (0.47–0.83)	0.028
50–54	1.64 (1.42–1.86)	<0.001	0.89 (0.68–1.10)	0.342
55–59	2.44 (2.25–2.63)	<0.001	1.44 (1.23–1.65)	0.015
60–64	3.06 (2.89–3.23)	<0.001	2.11 (1.98–2.24)	<0.001
65–69	3.46 (3.26–3.66)	<0.001	2.70 (2.51–2.89)	<0.001
70–74	3.71 (3.49–3.93)	<0.001	3.36 (3.17–3.55)	<0.001
75–79	3.40 (3.23–3.57)	<0.001	3.47 (3.29–3.65)	<0.001
80–84	3.17 (2.98–3.37)	<0.001	3.43 (3.25–3.61)	<0.001
85–89	3.61 (3.41–3.81)	<0.001	3.26 (3.07–3.45)	<0.001
90–94	2.52 (2.73–2.32)	<0.001	2.76 (2.59–2.93)	<0.001
Period effects				
1994	0.61 (0.40–0.83)	0.014	0.83 (0.62–1.04)	0.062
1999	0.72 (0.52–0.92)	0.040	0.78 (0.55–1.01)	0.055
2004	0.86 (0.70–1.02)	0.054	0.95 (0.74–1.16)	0.348
2009	1.11 (0.92–1.30)	0.200	1.07 (0.88–1.26)	0.482
2014	1.44 (1.26–1.62)	<0.001	1.18 (0.99–1.37)	0.052
2019	1.65 (1.44–1.86)	<0.001	1.29 (1.07–1.51)	0.033
Cohort effects				
1904–1908	3.92 (3.53–4.31)	<0.001	2.21 (1.89–2.53)	<0.001
1909–1913	3.21 (2.82–3.60)	<0.001	2.27 (1.90–2.54)	<0.001
1914–1918	2.60 (2.31–2.89)	<0.001	2.08 (1.76–2.41)	<0.001
1919–1923	2.16 (1.84–2.48)	<0.001	1.84 (1.50–2.19)	<0.001
1924–1928	1.98 (1.67–2.29)	<0.001	1.77 (1.45–2.10)	<0.001
1929–1933	1.73 (1.49–1.97)	<0.001	1.73 (1.41–2.06)	<0.001
1934–1938	1.52 (1.28–1.86)	<0.001	1.63 (1.37–1.89)	<0.001
1939–1943	1.36 (1.03–1.70)	0.042	1.53 (1.32–1.75)	<0.001
1944–1948	1.20 (0.89–1.52)	0.594	1.36 (1.15–1.57)	0.018
1949–1953	1.13 (0.88–1.38)	0.623	1.28 (1.07–1.49)	0.045
1954–1958	1.02 (0.79–1.25)	0.749	1.20 (0.98–1.42)	0.075
1959–1963	0.90 (0.68–1.13)	0.810	1.02 (0.82–1.22)	0.483
1964–1968	0.76 (0.53–0.99)	0.048	0.85 (0.68–1.03)	0.054
1969–1973	0.65 (0.44–0.86)	0.032	0.72 (0.50–0.94)	0.043
1974–1978	0.50 (0.28–0.73)	0.014	0.59 (0.38–0.80)	0.006
1979–1983	0.43 (0.21–0.66)	<0.001	0.51 (0.29–0.73)	<0.001
1984–1988	0.41 (0.20–0.62)	<0.001	0.41 (0.20–0.62)	<0.001
1989–1993	0.36 (0.15–0.57)	<0.001	0.34 (0.14–0.55)	<0.001
1994–1998	0.32 (0.11–0.54)	<0.001	0.33 (0.13–0.54)	<0.001
1999–2003	0.31 (0.09–0.53)	<0.001	0.37 (0.16–0.59)	<0.001
Deviance	1.39		0.35	
AIC	4.18		2.92	
BIC	−232.60		−233.64	

*Note*: RR denotes the relative risk of MM incidence and deaths in a particular age, period, or birth cohort relative to the average level of all age, period, or birth cohort combined.

Abbreviations: AIC, Akaike information criterion; BIC, Bayesian information criterion; CI, confidence interval; RR, relative risk.

**TABLE 2:** Sex‐specific relative risks of larynx cancer mortality in China due to age, period, and cohort effects.

Factors	Male		Female	
	RR (95% CI)	*p* value	RR (95% CI)	*p* value
Age				
20–24	0.05 (0.01–0.08)	<0.001	0.13 (0.02–0.25)	<0.001
25–29	0.06 (0.02–0.10)	<0.001	0.16 (0.03–0.30)	<0.001
30–34	0.09 (0.03–0.16)	<0.001	0.17 (0.03–0.32)	<0.001
35–39	0.21 (0.10–0.32)	<0.001	0.23 (0.05–0.41)	<0.001
40–44	0.47 (0.28–0.66)	<0.001	0.33 (0.09–0.57)	<0.001
45–49	0.82 (0.60–1.04)	0.078	0.54 (0.32–0.76)	<0.001
50–54	1.37 (1.04–1.70)	0.045	0.75 (0.58–0.92)	0.025
55–59	1.91 (1.68–2.14)	<0.001	1.14 (0.89–1.39)	0.442
60–64	2.39 (2.05–2.73)	<0.001	1.72 (1.46–1.98)	<0.001
65–69	2.97 (2.52–3.42)	<0.001	2.47 (2.09–2.85)	<0.001
70–74	3.76 (3.48–4.04)	<0.001	3.54 (3.01–4.07)	<0.001
75–79	3.98 (3.59–4.37)	<0.001	4.00 (3.52–4.48)	<0.001
80–84	4.66 (4.16–5.16)	<0.001	4.77 (4.18–5.36)	<0.001
85–89	6.07 (5.57–6.57)	<0.001	5.17 (4.57–5.77)	<0.001
90–94	5.39 (4.88–5.90)	<0.001	5.49 (4.86–6.12)	<0.001
Period				
1994	0.81 (0.61–1.01)	0.053	1.06 (0.96–1.16)	0.088
1999	0.87 (0.65–1.09)	0.118	0.91 (0.72–1.10)	0.748
2004	0.92 (0.74–1.11)	0.271	1.00 (0.82–1.18)	0.581
2009	1.03 (0.85–1.21)	0.555	1.00 (0.81–1.19)	0.580
2014	1.20 (0.98–1.42)	0.068	1.01 (0.85–1.17)	0.570
2019	1.25 (0.99–1.51)	0.051	1.01 (0.79–1.23)	0.603
Cohort				
1904–1908	3.61 (3.20–4.02)	<0.001	2.10 (1.76–2.44)	<0.001
1909–1913	3.20 (2.79–3.61)	<0.001	2.30 (1.94–2.66)	<0.001
1914–1918	2.76 (2.40–3.13)	<0.001	2.23 (1.87–2.59)	<0.001
1919–1923	2.45 (2.14–2.76)	<0.001	2.09 (1.71–2.48)	<0.001
1924–1928	2.34 (2.02–2.66)	<0.001	2.06 (1.68–2.44)	<0.001
1929–1933	2.11 (1.79–2.43)	<0.001	2.05 (1.70–2.40)	<0.001
1934–1938	1.89 (1.57–2.11)	<0.001	1.96 (1.66–2.26)	<0.001
1939–1943	1.69 (1.48–1.90)	<0.001	1.84 (1.54–2.14)	<0.001
1944–1948	1.46 (1.23–1.69)	<0.001	1.60 (1.29–1.91)	<0.001
1949–1953	1.32 (1.12–1.53)	0.001	1.45 (1.17–1.73)	<0.001
1954–1958	1.14 (0.95–1.33)	0.133	1.31 (1.08–1.54)	0.008
1959–1963	0.95 (0.78–1.12)	0.446	1.06 (0.83–1.29)	0.661
1964–1968	0.77 (0.59–0.96)	0.040	0.86 (0.62–1.11)	0.409
1969–1973	0.64 (0.43–0.86)	0.025	0.71 (0.49–0.93)	0.031
1974–1978	0.47 (0.25–0.69)	<0.001	0.55 (0.32–0.78)	<0.001
1979–1983	0.38 (0.17–0.60)	<0.001	0.45 (0.23–0.67)	<0.001
1984–1988	0.33 (0.14–0.52)	<0.001	0.34 (0.12–0.57)	<0.001
1989–1993	0.28 (0.06–0.50)	<0.001	0.27 (0.07–0.47)	<0.001
1994–1998	0.23 (0.03–0.43)	<0.001	0.24 (0.03–0.45)	<0.001
1999–2003	0.21 (0.01–0.42)	<0.001	0.27 (0.02–0.52)	<0.001
Deviance	0.92		0.25	
AIC	3.79		2.67	
BIC	−233.07		−233.74	

*Note*: RR denotes the relative risk of MM incidence and deaths in a particular age, period, or birth cohort relative to the average level of all age, period, or birth cohort combined.

Abbreviations: AIC, Akaike information criterion; BIC, Bayesian information criterion; CI, confidence interval; RR, relative risk.

Time period only had a significant effect on the incidence of larynx cancer for males. The RRs of larynx cancer incidence rate increased with time period in males from 0.61 (95% CI: 0.40 to 0.83) in 1994 to 1.65 (95% CI: 1.44 to 1.86) in 2019 (Supporting Information Figure S3A). Although the RRs increased from 0.81 (95% CI: 0.61 to 1.01) in 1994 to 1.25 (95% CI: 0.99 to 1.51) in 2019 for larynx cancer mortality in males (Supporting Information Figure S3B) and from 0.83 (95% CI: 0.62 to 1.04) in 1994 to 1.29 (95% CI: 1.07 to 1.51) in 2019 for larynx cancer incidence in females (Supporting Information Figure S3A), no statistical significance was found for these results. During the observation period, the RRs of deaths were stable for larynx cancer in females (Supporting Information Figure S3B).

For the birth cohort effects, later cohorts had a lower risk of larynx cancer incidence and mortality compared with the earlier cohorts. The RRs of incidence rates decreased from 3.92 (95% CI: 3.53 to 4.31) in the cohort 1904–1908 to 0.31 (95% CI: 0.09 to 0.53) in the cohort 1999–2003 for males, and from 2.21 (95% CI 1.89 to 2.53) in the cohort 1904–1908 to 0.37 (95% CI: 0.16 to 0.59) in the cohort 1999–2003 for females (Supporting Information Figure S4A). The RRs of death rates decreased from 3.61 (95% CI: 3.20 to 4.02) in the cohort 1904–1908 to 0.21 (95% CI: 0.01 to 0.42) in the cohort 1999–2003 for males, and from 2.10 (95% CI: 1.76 to 2.44) in the cohort 1904–1908 to 0.27 (95% CI: 0.02 to 0.52) in the cohort 1999–2003 for females (Supporting Information Figure S4B).

### 3.5 Prediction of larynx cancer incidence and deaths rates in 2019–2044 in China

**FIGURE 4:**
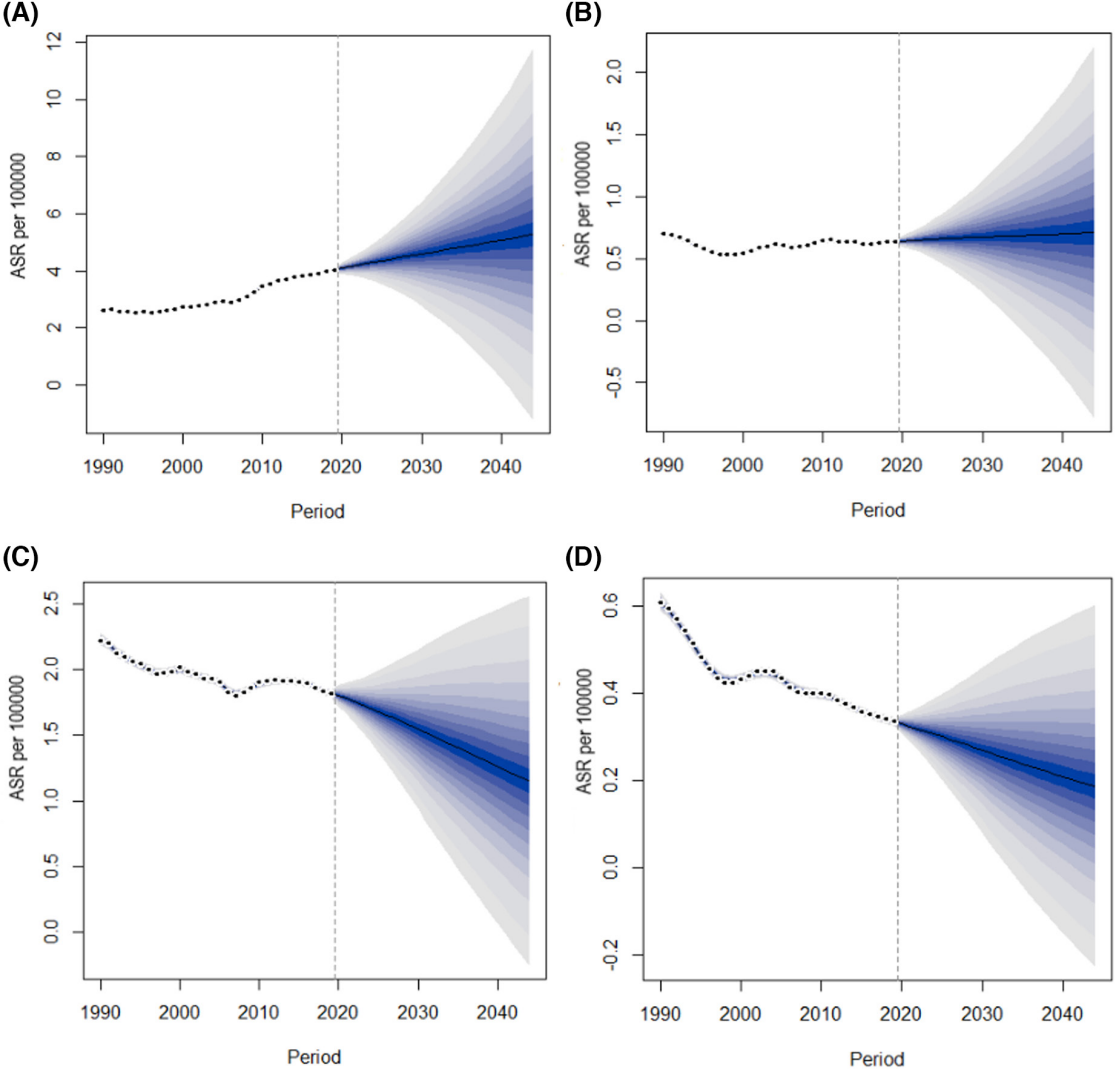
Projection of age‐standardized incidence and mortality rates of larynx cancer from 2020 to 2044 in China. (A) age‐standardized incidence rates in males; (B) age‐standardized incidence rates in females; (C) age‐standardized mortality rates in males; (D) age‐standardized mortality rates in females. ASR, age‐standardized rates.

The incidence rate of larynx cancer in China will continue to increase in the future, whereas the death rate will continue to decrease. According to the prediction, the age‐standardized incidence rates of larynx cancer will increase from 4.02 per 100,000 in 2019 to 5.27 per 100,000 in 2044 for males, and from 0.63 per 100,000 in 2019 to 0.71 per 100,000 in 2044 for females (Figure 4A,B). By contrary, the age‐standardized mortality rates of larynx cancer will decrease from 1.82 per 100,000 in 2019 to 1.15 per 100,000 in 2044 for males, and from 0.33 per 100,000 in 2019 for males to 0.19 per 100,000 in 2044 for females (Figure 4C,D). Although the increasing trend and decreasing trend of deaths of females are similar to those of males, the incidence and death rates of females are obviously lower than that of males.

## 4 DISCUSSION

Our study is the first to explore the long‐term trends of larynx cancer incidence and death rates in China between 1990 and 2019 by using the age–period–cohort framework. The increasing trend of age‐standardized incidence rates of larynx cancer for males from 1990 to 2019 in China may be related to increased exposure to various risk factors. Previous studies have revealed that smoking and alcohol use are the two most important risk factors for larynx cancer, which is consistent with our findings.^23^ According to a foreign pooled analysis including 19,600 cases and 25,566 controls, the risk of larynx cancer significantly increased among smokers than non‐smokers.^24^ Moreover, scholars revealed that drinking is a weak related factor of larynx cancer, but has significant synergistic effect with smoking.^25^ As a carcinogenic initiator or a promoter, ethanol may enhance the permeability of cells to other environmental carcinogens, especially tobacco smoke.^26^ In addition, long‐term occupational exposure to asbestos and sulfuric acid may lead to an increased risk of larynx cancer. In a case–control study of 1833 patients with head and neck squamous cell carcinoma in France, asbestos exposure increased the risk of larynx cancer, and this risk rosed with the length and severity of asbestos exposure.^27^ Besides, the stagnation of asbestos in the throat is more pronounced.^28^ Similar to asbestos fiber, exposure to sulfuric acid mist and smoking have a synergistic effect on larynx cancer.^29^

For both males and females, the overall trend of age‐standardized mortality rate of larynx cancer showed a decreasing trend from 1990 to 2019. This phenomenon may be related to the expansion of medical service coverage and the improvement of national health awareness, medical technology, and cancer screening and prevention system.

In terms of gender differences, the incidence and deaths rates of larynx cancer in China were significantly higher in males than in females, which may be due to the greater exposure to risk factors in males. Smoking is an important risk factor for respiratory tract tumors, including larynx cancer.^30^, ^31^, ^32^ China is a big country with high tobacco consumption, and Chinese males accounted for more than one third of the world's tobacco consumption,^33^ which was far higher than that in females, resulting in a higher incidence of larynx cancer in males than in females. Similarly, more males drink alcohol than females in China. The Scientific Research Report on Dietary Guidelines for Chinese Residents (2021) showed that the drinking rate was 64.5% in males and 23.1% in females, while the smoking rate for males was 50.5% and that for females was 11.8%.^34^ Besides smoking and alcohol use, sex hormones are also one of the risk factors of larynx cancer.^35^ Compared with normal controls, patients with larynx cancer have higher testosterone levels, but their estrogen levels are at the same levels, indicating that testosterone may promote the development of larynx cancer.^36^, ^37^ The majority of females with larynx cancer mostly occurs in menopause and old age, probably due to the decrease of estrogen level, suggesting that estrogen may have a protective effect on larynx cancer.^38^ Furthermore, the low long‐term exposure to harmful substances and an increase in HPV vaccination may account for the low incidence of larynx cancer in females.^39^

Our study revealed that the risk of incidence and deaths due to larynx cancer in China begins to significantly rise at the age of 50–69 years, and reaches the peak over the age of 70 years. The high risks of larynx cancer incidence and mortality among elderly population can be explained by the weakness of physiological function and immune system.^1^ With the acceleration of aging process in China, the seventh national census shows that the proportion of the population aged over 60 years has reached 18.70%, and the proportion of the population aged 65 years and above has reached 13.50%.^40^ The aging process will bring about a series of health problems, including the increased burden of malignant cancers. Therefore, we should adhere to the disease prevention and treatment strategy of early detection, early diagnosis, and early treatment, so as to effectively reduce the disease burden of laryngeal cancer.

With regard to the period effect, the risk of larynx cancer incidence in males increased significantly over time. This phenomenon can be attributed to increased exposure to unhealthy behaviors and harmful environmental factors. With the development of social economy and the quickening pace of life, males are facing more social pressure and life pressure than before, so they tend to smoke and drink to release their stress.^41^, ^42^ Similarly males are more likely to drink than females in China.^43^ Moreover, the exposure of occupational hazards gradually increases with the industrialization of society. In order to alleviate the disease burden caused by risk factors, some effective interventions should be taken. On the one hand, targeted education on smoking cessation and alcohol restriction is conducive to raise the awareness of disease prevention among high‐risk population, especially males and those who have been exposed to asbestos and sulfuric acid mist for the long term. Thus, improving alcohol use control strategies and adhering to current smoking control policies should become priorities for public health policies. On the other hand, reasonable occupational disease exposure protection should be strengthened, including the establishment of appropriate protection measures against materials and equipment that may cause occupational exposure hazards, as well as the establishment of occupational health monitoring records of workers and regular physical examination.

Regarding the cohort effect, the risk of larynx cancer incidence in the later birth cohorts was lower than that in the earlier birth cohorts. One of the possible reasons is the improvement of screening technology, which can detect precancerous lesions in time, thereby reducing the incidence of larynx cancer in the population. Advanced imaging methods have gradually played an important role in identifying early larynx cancer, such as narrowband imaging. Narrowband imaging is a highly specific and sensitive detection method that exhibits excellent accuracy in identifying precancerous and malignant lesions in the larynx.^44^, ^45^ Similarly, the decreasing risk of larynx cancer mortality in the late birth cohorts may be explained by the unprecedented advancement in the surgical and nonsurgical treatment technology.^23^ For advanced larynx cancer, compared with total laryngectomy, endoscopic partial laryngectomy combined with postoperative radiotherapy contributes to preserve part of organ functions as much as possible while treating cancer, thus reducing treatment complications and improving patients' survival rate and the quality of life.^46^

In the next 25 years, the incidence of larynx cancer in China will continue to rise, whereas the mortality rate will continue to decline, suggesting that larynx cancer patients will live longer with the disease. In this regard, it is important to improve the quality of life of the patients with larynx cancer. First, clinicians should improve the treatment techniques to reduce the side effects of treatment, such as the preservation or reconstruction of laryngeal function during surgical resection, and the reduction of adverse reactions of radiotherapy.^2^ Second, medical staff should strengthen psychological counseling and functional exercise for patients with larynx cancer after surgery.^47^ Finally, supportive social interaction, such as listening, responding, and encouragement, can help to improve the self‐efficacy and self‐confidence, relieve the depression and pain, and promote functional recovery, thereby improving the quality of life of the patients.^48^ Moreover, it is necessary to further explore the risk factors attributed to larynx cancer, especially the changeable risk factors, and develop individualized risk quantification strategies, so as to encourage people to change the adverse lifestyle, early detect precancerous lesions, and reduce the burden of larynx cancer. Our study has some inevitable limitations. First, due to lack of the epidemiological survey data of larynx cancer in China, and great heterogeneity among the studies, the estimation of GBD may be inconsistent with the actual data. Although GBD collects as many published and unpublished data as possible, the quantity and quality of data about larynx cancer remain limited. For example, the data used in the GBD was obtained from cancer registries, which may not cover all cases of larynx cancer in China. Second, larynx cancer is mainly divided into well‐differentiated squamous cell carcinomas, while chondrosarcomas, leiomyosarcomas, and melanomas. However, due to the insufficient data in the GBD study, we are unable to conduct further subgroup analysis based on the clinical features of larynx cancer. Third, due to insufficient information, we were unable to analyze the burden of larynx cancer in China by stratification, such as province, economic development, and ethnicity. Finally, although the IE methods used in this study has the characteristics of effectiveness, asymptotic, and optimal estimation, its theoretical basis is complex, which cannot explain the practical significance of parameter estimation.

## 5 CONCLUSION

The incidence rate of larynx cancer in China shows an upward trend for males during the past three decades and will continue to increase in the next 25 years. The mortality rate of larynx cancer shows a downward trend during the observation period and will continue to decline until 2044. Age and cohort effect have significant effects on the burden of larynx cancer with a high risk of incidence and mortality due to larynx cancer in elderly population and early‐born cohorts. Therefore, understanding of the risk factors and disease pattern of larynx cancer and establishing effective interventions to reduce the burden of larynx cancer are needed.

## AUTHOR CONTRIBUTIONS

**Enlin Ye:** Conceptualization (equal); data curation (equal); formal analysis (equal); funding acquisition (equal); investigation (equal); methodology (equal); project administration (equal); resources (equal); software (equal); supervision (equal); validation (equal); visualization (equal); writing – original draft (equal); writing – review and editing (equal). **Jiasheng Huang:** Conceptualization (equal); data curation (equal); formal analysis (equal); funding acquisition (equal); investigation (equal); methodology (equal); project administration (equal); resources (equal); software (equal); supervision (equal); validation (equal); visualization (equal); writing – original draft (equal); writing – review and editing (equal). **Jia Wang:** Data curation (equal); resources (equal); software (equal). **Yumei Zhao:** Data curation (equal); resources (equal); software (equal). **Dongdong Niu:** Conceptualization (equal); visualization (equal). **Jie Liu:** Conceptualization (equal); validation (equal). **Xueying Huang:** Conceptualization (equal); validation (equal). **Suru Yue:** Conceptualization (equal); visualization (equal). **Xuefei Hou:** Data curation (equal); resources (equal); software (equal). **Jiayuan Wu:** Conceptualization (equal); data curation (equal); formal analysis (equal); funding acquisition (equal); investigation (equal); methodology (equal); project administration (equal); resources (equal); software (equal); supervision (equal); validation (equal); visualization (equal); writing – review and editing (equal).

## CONFLICT OF INTEREST STATEMENT

The authors declare no conflict of interest.

## ETHICAL APPROVAL STATEMENT

This article does not contain any studies with human participants or animals performed by any of the authors. The review and approval were not required for this research by an institutional review board or ethics committee because this study used a public database with epidemiological data and the article does not contain any studies with human participants or animals performed by any of the authors.

## Supporting information


Figure S1.

Figure S2.

Figure S3.

Figure S4.

## Data Availability

Data sharing is not applicable to this article as no new data were created or analyzed in this study.
