# Supplementary figures and images for: Trend and projection of larynx cancer incidence and mortality in China from 1990 to 2044: A Bayesian age–period–cohort modeling study
Source: Cancer Med. 2023 Jun 12;12(15):16517–30. doi: 10.1002/cam4.6239 (PMC10469639; doi:10.1002/cam4.6239)

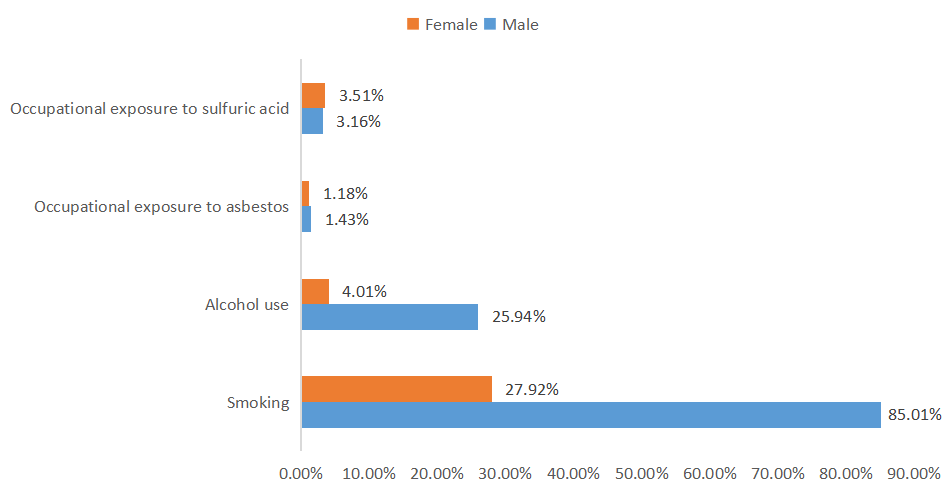

Supplement: Supplementary file 1 — Figure S1. [file CAM4-12-16517-s002.tiff]

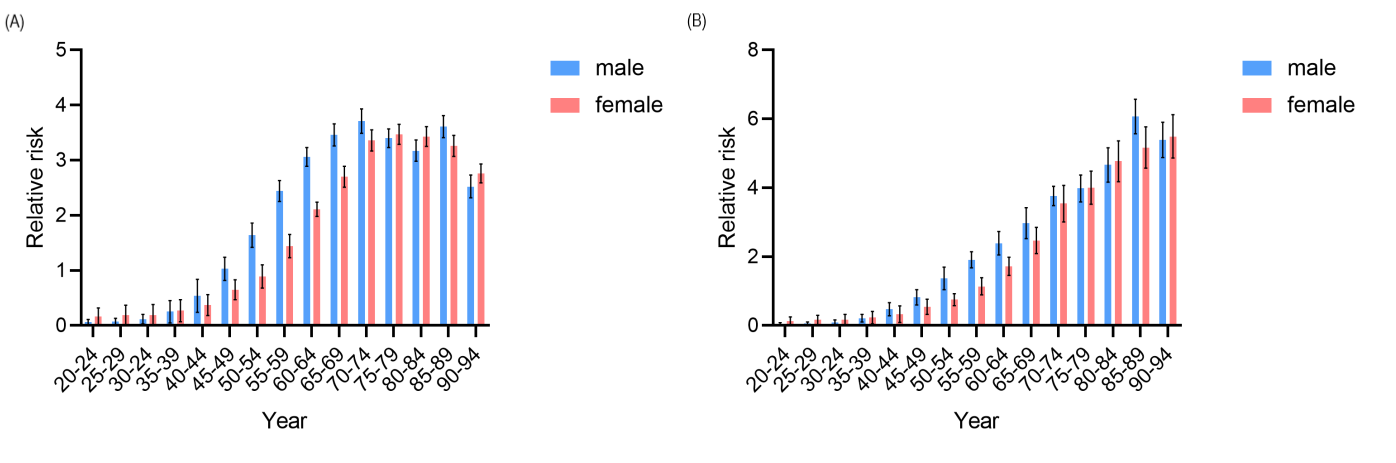

Supplement: Supplementary file 2 — Figure S2. [file CAM4-12-16517-s001.tiff]

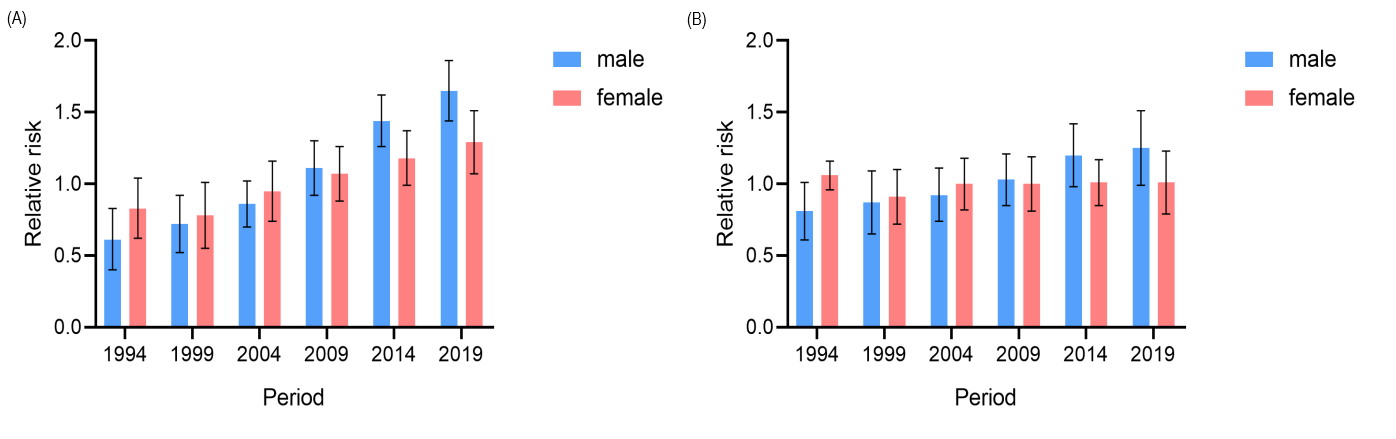

Supplement: Supplementary file 3 — Figure S3. [file CAM4-12-16517-s003.tiff]

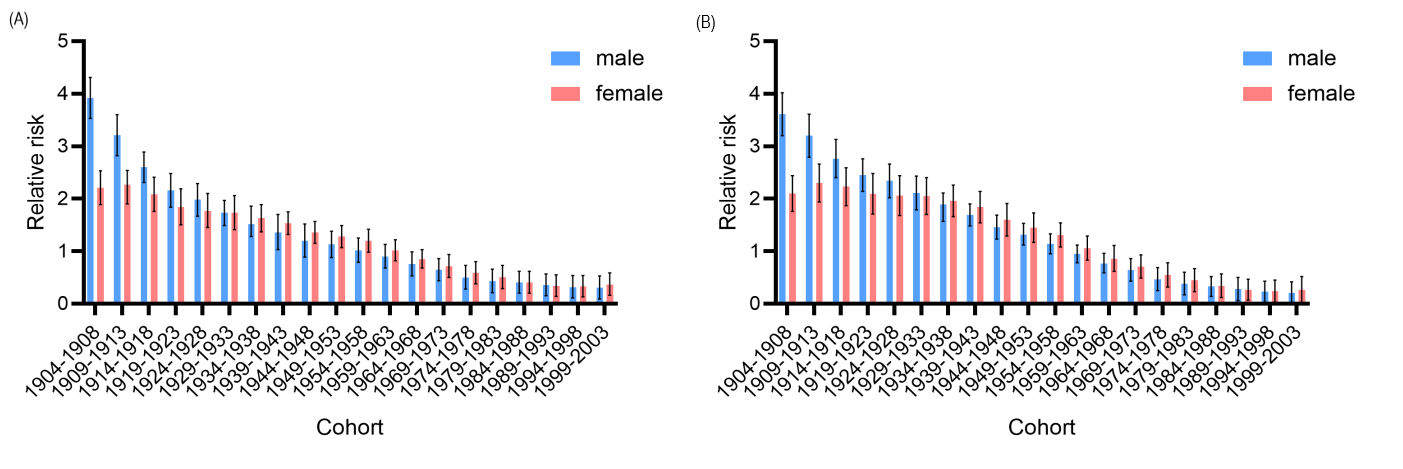

Supplement: Supplementary file 4 — Figure S4. [file CAM4-12-16517-s004.tiff]
